# Supplementary material for: Toward an integrated model of dietary behavior: Social determinants and adherence to the mediterranean diet
Source: PLoS One. 2025 Dec 17;20(12):e0328853. doi: 10.1371/journal.pone.0328853 (PMC12711094; doi:10.1371/journal.pone.0328853)
Supplement: Attachment — Submitted filename: Response to Reviewers-.docx [file pone.0328853.s003.docx]

Dear Editor and Reviewers,

Thank you for your thoughtful feedback—our revisions have benefited substantially from your comments.

We confirm full compliance with PLOS ONE style (incl. file naming) and have uploaded Response to Reviewers, Revised Manuscript with Track Changes, and Manuscript. The funders had no role in study design, data collection and analysis, decision to publish, or preparation of the manuscript. We have shared the complete minimal data set

**Reviewer #1**

**1.Biases and validity concerns**

We added a short integrative paragraph in the *limitations* section that directly addresses the methodological issues raised and clarifies our approach and rationale for key design choices. Specifically, the new paragraph is as follows.

- discusses the recruitment method based on social media that does not rely on probability, along with the consequences for selection bias and the digital divide;
- acknowledges the female-skewed sample and limited age diversity, clarifying that we intentionally did not apply post-hoc adjustments to observe natural interest by gender and that comparisons are descriptive;
- notes the self-reported nature of measures and how we mitigated recall risk.
- frames the web-based component as one part of a broader, ongoing program, positioning the current results as exploratory.

**2. Handling of missing data**

In the same integrative paragraph, we clarified that we used pairwise deletion, and reported item-level valid *N* for transparency. This explains our analytic choices and their rationale without overextending claims.

**3. Introduction is lengthy**

We streamlined the *Introduction*, focusing on the rationale, objectives, and key hypotheses and removing non-essential theoretical digressions.

**4. Operationalization of Mediterranean-diet adherence**

We have now stated concisely how adherence is defined and scored, so that readers can grasp the index without a long methodological detour.

**Reviewer #2**

We appreciate this positive appraisal. We have made minor editorial refinements and incorporated the clarifications above to enhance transparency and interpretability.
